# Sports activity and changes in physical fitness of Polish children and adolescents: OSF study

**DOI:** 10.3389/fped.2022.976943

**Published:** 2022-08-11

**Authors:** Joanna Baj-Korpak, Kamil Zaworski, Marian J. Stelmach, Piotr Lichograj, Marek Wochna

**Affiliations:** ^1^Department of Health Sciences, John Paul II University of Applied Sciences, Biala Podlaska, Poland; ^2^Department of Technical Sciences, John Paul II University of Applied Sciences, Biala Podlaska, Poland; ^3^Polish Athletic Association, Warsaw, Poland

**Keywords:** sports activity, physical activity, physical fitness, Body Mass Index (BMI), Ponderal Index (PI)

## Abstract

**Background:**

Physical activity promotion programs for children and adolescents should constitute the basis of any national health policy aiming to improve physical fitness, which is a significant health indicator in children, adolescents, adults as well as elderly persons.

**Methods:**

The study included 1,230 children and adolescents aged 8–16 years (60.1% of girls) from Poland. Five hundred and twenty-seven persons covered by the survey (42.8%) participated in physical activity promotion called “Athletics for All!” (AFA) as an experimental group and 703 peers not participating in any forms of physical extra-curricular activities as a control group. Participants were measured for basic anthropometric parameters and body mass indices were calculated: Body Mass Index (BMI) and Ponderal Index (PI). Evaluation of physical fitness was made using the OSF Test (3 × 10 m shuttle run, standing broad jump, 1 kg medicine ball overhead throw, 4-min run). Comparative analysis between the experimental and control groups was carried out using the *T*-test for independent samples. Analysis of correlations between quantitative variables was performed with Pearson's *r* coefficient.

**Results:**

Statistically significant differences in all the tests were noted between the experimental group (AFA) and the control group in favor of the former one. Taking into account reference ranges of BMI created according to the latest Polish norms, statistically significant differences were noted between the subgroups in all physical fitness tests. As far as PI is concerned, in the AFA group significant differences were noted in all physical fitness tests between subgroups created in accordance with the aforementioned norms.

**Conclusion:**

The findings of our study show that it is necessary to introduce physical activity promotion programs for children and adolescents. Such programs should constitute the basis of national health policy aiming at improving physical fitness among young people.

## Introduction

Increased physical activity (PA) produces numerous health-related benefits that are particularly important for children and adolescents. These include improved physical fitness and mental health, enhanced cardiovascular fitness and metabolism as well as better developed musculoskeletal system (1–4). Moreover, PA may exert a positive influence on cognitive abilities and learning outcomes (5). Increased PA also constitutes the main element of prevention of obesity and metabolic diseases (including type 2 diabetes) (2). Due to the epidemic of obesity among children worldwide, activities aimed at health promotion and taking up physical activity are extremely significant (6).

According to the latest guidelines of the World Health Organization (WHO), it is recommended that children and adolescents aged 5–17 should perform moderate-to-vigorous physical activity (MVPA) for at least 60 min every day. At the same time, they should do vigorous PA as well as muscle- and bone-strengthening exercises at least three times a week (7).

Regular PA is an indication of the acquired health competences: knowledge, skills, beliefs, attitudes and needs associated with health (8). Unfortunately, a large proportion of children and adolescents do not follow recommendations regarding PA (9). Sedentary activities, i.e., activities performed in a sitting or lying position with energy expenditure of ≤1.5 METs (Metabolic Equivalent of Tasks) constitute a common element of lifestyle in contemporary societies all over the world (10). Since 2020, COVID-19 pandemic has brought about changes in the functioning of many areas of economy and education (working from home and distance learning), which has led to a rise in sedentary behaviors (11). Therefore, there is an urgent need to increase PA particularly among children and adolescents. It is schools as well as external organizations that should promote PA in this group (3).

Physical fitness (PF) can be defined in a variety of ways. Most often, it is described as an ability to perform body movements vigorously, without excessive fatigue and with enough energy to cope with different life situations. Thus, this term encompasses endurance of the respiratory system and skeletal muscles, strength and power of skeletal muscles, speed, flexibility, agility, balance, reaction time and body composition (12, 13). PF level is conditioned by a number of factors. It depends on both genetic factors and regular PA (14). In the case of children and adolescents, it is particularly important to implement PA with exercises aimed at developing motor abilities that will ensure effective muscle performance in adult life (15). Blair et al. (16, 17) reported that higher levels of PF in adults reduce the risk of cardiovascular diseases and cancer significantly and delay mortality caused by any factors. In turn, lower PF levels increase such risk both in men and in women.

In the literature we can come across numerous studies on measurement and evaluation of PF in different populations and social groups (18–23). The use of different tests is a necessary form of assessing and monitoring motor ability levels (24). It is based on testing motor abilities in a simple and intelligible manner, and each test ought to be reliable and valid (25).

The development of batteries of fitness tests began in the USA after the publication of Kraus regarding the comparison of PF between American children and their European counterparts in the 1950s (26). The first European study based on American methodology was carried out in the 1960s in Belgium and the Netherlands (27). In Poland, the most common tests are Denisiuk's Physical Fitness Test, Trzesniowski's Physical Fitness Measure, International Physical Fitness Test, Eurofit and Zuchora's Index of Physical Fitness (25).

Physical fitness of children and adolescents is the main area of interest of teachers, doctors, physical therapists and parents. Different sets of tests are used to assess PF (28, 29)—teachers' area of interest is the assessment of PF levels (performance progression), medics' area of interest is the health-related aspect (recovery, rehabilitation progression), while parents' area of interest is the general wellbeing of children.

PF may be measured accurately with the use of laboratory tests; however, due to the need to employ proper measurement instruments and because of high costs and time limitations, it is still impossible to conduct such examinations on the whole population. Field tests are easy to carry out, can be performed with little equipment, they are cheap and can be used with a larger number of people and over a longer period of time (30). Three components are assessed most often: (1) anthropometric parameters, (2) physical capacity parameters, (3) motor abilities.

Anthropometric parameters that are measured most frequently include body height and mass as well as BMI or PI (31). Physical capacity parameters are usually measured using running tests due to their simplicity and accessibility. Motor abilities are assessed with the use of specially designed tests with particular components included in them.

Eurofit, FitnessGram, and Alpha-fit are the most commonly used batteries of tests (32). Monitoring PF levels in children and adolescents is significant not only because PA is crucial in maintaining health but also owing to the fact that it facilitates the selection of talented individuals for particular sports (33).

The aim of the study was to assess differences in PF of children and adolescents participating in Athletics for All program (AFA) and those not participating in any extra-curricular sports activities.

The study sought to determine whether the implementation of programs promoting PA such as AFA may improve PF of young individuals and whether it improves anthropometric parameters (BMI, PI).

## Materials and methods

### Participants

Since 2014, the Polish Athletic Association (PAA) has been supervising the program of physical activity promotion called “Athletics for All!” (AFA)^1^, whose aim is to promote athletics as “the first choice” sport among children and adolescents. Sports activities within the program are conducted by qualified athletic coaches and instructors. The program is financed by the Ministry of Sport and Tourism, local governments and external sponsors. AFA includes over 600 training groups in Poland. Training sessions are held taking into account the age of the participants (grades 1–8 of primary school) and their level of sports advancement. The most accomplished children may continue their careers in the so-called Centers of Oriented Training (COTs). In COTs, training is more advanced; adolescents take part in sports competitions and training camps and undergo physical fitness tests regularly. In the youngest group (grades 1–4 of primary school, i.e., 6–10 years of age), 90-min training sessions are held twice a week. In grades 5–8 (10–14 years of age), 90-min sessions are held three times a week, while in COTs (13–17 years of age) 90-min sessions take place 5 times a week^2^.

The study included 1,230 young individuals from Poland, i.e., 527 children and adolescents (42.8%) participating in AFA (experimental group) and 703 individuals not participating in any forms of physical extra-curricular activities (control group). Average age of the respondents (M) was 12.21 years (SD = 1.11)—the age of the study participants was calculated in decimal values taking into consideration date of birth and date of examination. Girls constituted a larger proportion of the studied population (60.1%)—Table 1.

**Table 1:** Characteristics of the studied population (*N* = 1,230).

**Group**	**Variable**	**Gender**	***N***	**Mean**	**Standard deviation**
Total	Age (years)	F	739	12.28	1.18
		M	491	12.11	0.99
	Body height (cm)	F	739	156.26	9.31
		M	491	157.66	10.78
	Body mass	F	739	47.92	11.31
		M	491	48.52	12.97
	BMI	F	739	19.43	3.41
		M	491	19.28	3.47
	PI	F	739	12.43	2.05
		M	491	12.23	2.04
AFA	Age (years)	F	302	12.32	0.82
		M	225	12.30	0.99
	Body height (cm)	F	302	158.02	7.89
		M	225	159.32	10.36
	Body mass	F	302	47.35	9.64
		M	225	48.03	11.19
	BMI	F	302	18.82	2.80
		M	225	18.72	2.79
	PI	F	302	11.91	1.65
		M	225	11.76	1.64
Control	Age (years)	F	437	12.24	1.37
		M	266	11.95	0.97
	Body height (cm)	F	437	155.05	10.01
		M	266	156.26	10.96
	Body mass	F	437	48.31	12.33
		M	266	48.94	14.31
	BMI	F	437	19.85	3.72
		M	266	19.75	3.91
	PI	F	437	12.80	2.22
		M	266	12.63	2.26

The study was carried out in April and May 2017 according to strictly defined rules (in compliance with the Evaluation of Physical Fitness test—OSF). Prior to the study, AFA coaches had been trained in terms of the study protocol. Each participant provided a written informed consent signed by their parents or legal guardians to take part in the OSF test and have their body height and mass measured.

### Anthropometric measurements

Measurements were made using properly calibrated equipment. Each measurement was made twice in the same conditions. If the difference between the first and the second measurement was 300 g or more for body mass and 5 mm or more for body height, the third measurement was performed.

Body height was measured with an accuracy of 1 mm using SECA 213 stadiometer. Each participant stood barefoot with their hips and arms perpendicular to the longitudinal axis, knees together, arms at the side and the head in the Frankfurt plane.

Body mass was measured using SECA 875 scales in accuracy class 3 (200 g). The following indices were calculated: (1) Body Mass Index (BMI)—body mass in kilograms divided by the square of body height in meters, (2) Ponderal Index (PI)—body mass in kilograms divided by the cube of body height in meters.

### Evaluation of physical fitness

Evaluation of physical fitness was made using the OSF (Evaluation of Physical Fitness) test that consists of four validated tests:

- 3 × 10 m shuttle run (speed test),
- standing broad jump (power test),
- 1 kg medicine ball overhead throw (strength test),
- 4-min run (endurance test),^3^

Test performance was preceded by a 5-min warm-up. Participants were familiarized with the tasks both theoretically and practically (task performance during the warm-up).

The OSF test was carried out indoors (sports hall) or outdoors (sports field) on natural or synthetic surfaces. All the testing stations had been set up prior to the commencement of the test. Each station was supervised by two persons, i.e., an AFA coach who was responsible for making measurements and a person who recorded the results. Throughout the test, participants were wearing sports clothes (shorts, a T-shirt, sports shoes).

The results of individual fitness tests were standardized and converted into points on a scale from 1 to 100, taking into account the age and gender of the participants, with a higher number of points indicating a higher fitness level.

### Statistical analysis

Statistical analysis of results was performed using SPSS 17.0 (Softonic, USA). To calculate qualitative data, two correlation coefficients based on the Chi-squared test were employed, i.e., Phi and V Kramer. For variables on ordinal scales, Kendall's Tau-b and Tau-c were applied.

Comparative analysis between the experimental and con groups was carried out using the *T*-test for independent samples.

Quantitative variables were described in terms of the parametric distribution (checked with the Shapiro-Wilk test and the Kolmogorov-Smirnov test) taking into account such descriptive characteristics as mean (M) and standard deviation (SD). One-way ANOVA was used for comparisons of equally numbered groups. If the homogeneity of variance was disturbed, the Games-Howell test was used for *post-hoc* comparisons.

Analysis of correlations between quantitative variables was performed with Pearson's *r* coefficient.

Statistical significance was set at α = 0.05. The Bioethics Committee of Warsaw University of Life Sciences, Faculty of Human Nutrition and Consumer Sciences (Resolution No. 16/2017) approved the protocol in accordance with the Declaration of Helsinki.

## Results

The AFA group included 523 participants (mean age of 12.31 years), while the control group consisted of 703 individuals (mean age of 12.14 years).

Statistically significant differences were noted between the AFA group and the control group in all the fitness tests (Table 2) and body mass indices (Table 3).

**Table 2:** Differences in physical fitness levels measured with the use of OSF.

**Fitness test**	**Group**	**Mean**	**SD**	**Standard error**	***t***	***p*-value**	**Difference in means**	**Standard error of difference in means**	**95% CI**	
									**Lower**	**Upper**
3 × 10 m shuttle run (s)	AFA	8.32	0.61	0.03	−21.01	0.001^***^	−0.87	0.04	−0.95	−0.79
	Control	9.19	0.84	0.03						
Standing broad jump (m)	AFA	1.82	0.36	0.02	5.95	0.001^***^	0.24	0.04	0.16	0.32
	Control	1.58	0.87	0.03						
Medicine ball throw (m)	AFA	7.82	1.79	0.08	14.11	0.001^***^	1.38	0.10	1.19	1.58
	Control	6.44	1.58	0.06						
4-min run (m)	AFA	815.74	95.20	4.15	17.07	0.001^***^	98.12	5.75	86.84	109.40
	Control	717.62	103.07	3.89						

*** Statistical significance p-value ≤ 0.001.

**Table 3:** Differences in body mass indices between AFA and Control groups.

**Group**		***N***	**Mean**	**SD**	**Difference in means**	**95% CI**		***p*-value**	**Cohen's D**
						**Lower**	**Upper**		
BMI	AFA	527	18.78	2.79	−1.03	−1.40	−0.67	0.001^***^	−0.30
	Control	703	19.81	3.79					
PI	AFA	527	11.84	1.64	−0.89	−1.11	−0.67	0.001^***^	−0.44
	Control	703	12.74	2.24					

*** Statistical significance p-value ≤ 0.001.

Statistically significant differences in all the tests were noted between the AFA group and the control group in favor of the former one. Taking into account BMI as a differentiating variable, a positive correlation was found in 3 × 10 m and the medicine ball throw, while a negative correlation was observed in 4-min run. Moreover, in the AFA group a positive correlation was noted between PI, 3 × 10 m and the medicine ball throw, whereas a negative correlation was found between PI and standing broad jump as well as 4-min run. In the control group, similar correlations were noted taking into consideration age (with the exception of 4-min run) and BMI. In the case of PI, a positive correlation occurred in 3 × 10 m shuttle run and the medicine ball throw, while a negative correlation was noted in 4-min run (Table 4).

**Table 4:** Correlations of physical fitness levels with age and anthropometric indices.

**Fitness test**	**Pearson's correlation**	**AFA group**			**Control group**		
		**Age**	**BMI**	**PI**	**Age**	**BMI**	**PI**
3 × 10 m shuttle run (s)	*r*	−0.273	0.115	0.241	−0.193	0.120	0.210
	*p*	0.001^***^	0.008^**^	0.001^***^	0.001^***^	0.001^***^	0.001^***^
Standing broad jump (m)	*r*	0.244	−0.027	−0.148	0.114	−0.030	−0.049
	*p*	0.001^***^	0.541	0.001^***^	0.003^**^	0.424	0.192
Medicine ball throw (m)	*r*	0.418	0.347	0.127	0.470	0.319	0.125
	*p*	0.001^***^	0.001^***^	0.004^**^	0.001^***^	0.001^***^	0.001^***^
4-min run (m)	*r*	0.28	−0.253	−0.335	0.059	−0.341	−0.358
	*p*	0.001^***^	0.001^***^	0.001^***^	0.116	0.001^***^	0.001^***^

** Statistical significance p-value ≤ 0.01.

*** Statistical significance p-value ≤ 0.001.

Statistically significant differences were noted between girls and boys in all physical fitness tests in the AFA group and in almost all tests in the control group (with the exception of standing broad jump) (Table 5).

**Table 5:** Differences in the OSF test results taking into account gender.

**AFA group**										
**Fitness test**	**Group**	**Mean**	**SD**	**Standard error**	***t***	***p*-value**	**Difference in means**	**Standard error of difference in means**	**95% CI**	
									**Lower**	**Upper**
3 × 10 m shuttle run (s)	F	8.46	0.57	0.03	6.46	0.001^***^	0.33	0.05	0.23	0.43
	M	8.13	0.60	0.04						
Standing broad jump (m)	F	1.78	0.42	0.02	−2.79	0.006^**^	−0.09	0.03	−0.15	−0.03
	M	1.87	0.27	0.02						
Medicine ball throw (m)	F	7.49	1.48	0.09	−4.80	0.001^***^	−0.78	0.16	−1.09	−0.46
	M	8.27	2.06	0.14						
4-min run (m)	F	794.94	91.81	5.28	−6.00	0.000^***^	−48.71	8.12	−64.65	−32.76
	M	843,65	92,68	6,18						
**Control group**										
**Test**	**Group**	**Mean**	**SD**	**Standard error**	***t***	***p*** **-value**	**Difference in means**	**Standard error of difference in means**	**95% CI**	
									**Lower**	**Upper**
3 × 10 m shuttle run (s)	F	9.33	0.84	0.04	5.96	0.001^***^	0.38	0.06	0.26	0.51
	M	8.95	0.79	0.05						
Standing broad jump (m)	F	1.57	1.08	0.05	−0.44	0.660	−0.03	0.07	−0.16	0.10
	M	1.60	0.25	0.02						
Medicine ball throw (m)	F	6.24	1.50	0.07	−4.43	0.001^***^	−0.54	0.12	−0.77	−0.30
	M	6.77	1.66	0.10						
4-min run (m)	F	694.16	92.55	4.43	−7.78	0.001^***^	−61.98	7.96	−77.63	−46.34
	M	756.15	107.93	6.62						

** Statistical significance p-value ≤ 0.01.

*** Statistical significance p-value ≤ 0.001.

Taking into account reference ranges of BMI created according to the norms developed in the OLAF study (34), statistically significant differences were noted between the subgroups in all physical fitness tests. In the control group, significant differences between BMI subgroups were found in 3 × 10 m shuttle run, medicine ball throw and 4-min run (Tables 6, 6.1).

**Table 6:** Physical fitness test results taking into account BMI.

**Fitness test**	**BMI**	***N***	**Mean**	**SD**	**Standard error**	**95% CI**		**Normality of distribution tests**		***p*-value**
						**Lower**	**Upper**	**Kolmogorov-Smirnov**	**Shapiro-Wilk**	
**AFA group**										
3 × 10 m shuttle run (s)	Underweight	272	8.32	0.63	0.04	8.24	8.39	0.001	0.001	0.001^***^
	Norm	239	8.26	0.54	0.04	8.19	8.33	0.001	0.001	
	Overweight and obesity	16	9.15	0.51	0.13	8.88	9.42	0.115	0.257	
Standing broad jump (m)	Underweight	272	1.81	0.44	0.03	1.76	1.86	0.001	0.001	0.002^**^
	Norm	239	1.86	0.25	0.02	1.82	1.89	0.084	0.095	
	Overweight and obesity	16	1.53	0.18	0.04	1.43	1.62	0.149	0.736	
Medicine ball throw (m)	Underweight	272	7.24	1.61	0.09	7.05	7.43	0.004	0.001	0.001^***^
	Norm	239	8.45	1.77	0.11	8.22	8.67	0.200^a^	0.007	
	Overweight and obesity	16	8.44	1.76	0.44	7.49	9.38	0.200^a^	0.757	
4-min run (m)	Underweight	272	823.45	98.33	5.96	811.71	835.19	0.001	0.001	0.001^***^
	Norm	239	816.86	83.82	5.42	806.18	827.54	0.200^a^	0.597	
	Overweight and obesity	16	667.81	85.03	21.26	622.50	713.12	0.200^a^	0.296	
**Control group**										
3 × 10 m shuttle run (s)	Underweight	295	9.15	0.93	0.05	9.04	9.26	0.001	0.001	0.001^***^
	Norm	338	9.14	0.73	0.04	9.06	9.22	0.011	0.002	
	Overweight and obesity	70	9.58	0.85	0.10	9.38	9.78	0.200^a^	0.681	
Standing broad jump (m)	Underweight	295	1.56	0.25	0.01	1.53	1.59	0.003	0.098	0.071
	Norm	338	1.64	1.22	0.07	1.51	1.77	0.001	0.001	
	Overweight and obesity	70	1.39	0.24	0.03	1.34	1.45	0.200^a^	0.245	
Medicine ball throw (m)	Underweight	295	5.85	1.45	0.08	5.68	6.02	0.019	0.001	0.001^***^
	Norm	338	6.80	1.48	0.08	6.64	6.96	0.001	0.001	
	Overweight and obesity	70	7.17	1.74	0.21	6.76	7.59	0.200^a^	0.764	
4-min run (m)	Underweight	295	745.23	99.83	5.81	733.79	756.67	0.005	0.001	0.001^***^
	Norm	338	712.6	93.22	5.07	702.68	722.63	0.001	0.001	
	Overweight and obesity	70	625.23	105.49	12.61	600.07	650.38	0.052	0.001	

a Lower limit of actual significance.

** Statistical significance p-value ≤ 0.01.

*** Statistical significance p-value ≤ 0.001.

**Table 6.1:** Significance of differences in physical fitness in relation to BMI confirmed by *post-hoc* test (Games-Howell test).

**Dependent variable**			**Difference in means**	**Standard error**	**Relevance**	**95% CI**	
						**Lower**	**Upper**
**AFA group**							
3 × 10 m shuttle run (s)	Underweight	Norm	0.06	0.05	0.525	−0.07	0.18
		Overweight and obesity	−0.83^*^	0.13	0.000	−1.17	−0.49
	Norm	Underweight	−0.06	0.05	0.525	−0.18	0.07
		Overweight and obesity	−0.88^*^	0.13	0.000	−1.22	−0.55
	Overweight and obesity	Underweight	0.83^*^	0.13	0.000	0.49	1.17
		Norm	0.88^*^	0.13	0.000	0.55	1.22
Standing broad jump (m)	Underweight	Norm	−0.05	0.03	0.304	−0.12	0.03
		Overweight and obesity	0.28^*^	0.05	0.000	0.15	0.41
	Norm	Underweight	0.05	0.03	0.304	−0.03	0.12
		Overweight and obesity	0.33^*^	0.05	0.000	0.21	0.45
	Overweight and obesity	Underweight	−0.28^*^	0.05	0.000	−0.41	−0.15
		Norm	−0.33^*^	0.05	0.000	−0.45	−0.21
Medicine ball throw (m)	Underweight	Norm	−1.21^*^	0.15	0.000	−1.56	−0.85
		Overweight and obesity	−1.20^*^	0.45	0.043	−2.36	−0.04
	Norm	Underweight	1.21^*^	0.15	0.000	0.85	1.56
		Overweight and obesity	0.01	0.46	1.000	−1.16	1.18
	Overweight and obesity	Underweight	1.20^*^	0.45	0.043	0.04	2.36
		Norm	−0.01	0.46	1.000	−1.18	1.16
4-min run (m)	Underweight	Norm	6.59	8.06	0.692	−12.35	25.53
		Overweight and obesity	155.64^*^	22.08	0.000	99.14	212.14
	Norm	Underweight	−6.59	8.06	0.692	−25.53	12.35
		Overweight and obesity	149.05^*^	21.94	0.000	92.77	205.32
	Overweight and obesity	Underweight	−155.64^*^	22.08	0.000	−212.14	−99.14
		Norm	−149.05^*^	21.94	0.000	−205.32	−92.77
**Control group**							
3 × 10 m shuttle run (s)	Underweight	Norm	0.01	0.07	0.98	−0.15	0.17
		Overweight and obesity	−0.43^*^	0.12	0.00	−0.70	−0.16
	Norm	Underweight	−0.01	0.07	0.98	−0.17	0.15
		Overweight and obesity	−0.44^*^	0.11	0.00	−0.70	−0.18
	Overweight and obesity	Underweight	0.43^*^	0.12	0.00	0.16	0.70
		Norm	0.44^*^	0.11	0.00	0.18	0.70
Standing broad jump (m)	Underweight	Norm	−0.09	0.07	0.41	−0.25	0.07
		Overweight and obesity	0.16^*^	0.03	0.00	0.09	0.24
	Norm	Underweight	0.09	0.07	0.41	−0.07	0.25
		Overweight and obesity	0.25^*^	0.07	0.00	0.08	0.42
	Overweight and obesity	Underweight	−0.16^*^	0.03	0.00	−0.24	−0.09
		Norm	−0.25^*^	0.07	0.00	−0.42	−0.08
Medicine ball throw (m)	Underweight	Norm	−0.95^*^	0.12	0.00	−1.23	−0.68
		Overweight and obesity	−1.32^*^	0.22	0.00	−1.85	−0.79
	Norm	Underweight	0.95^*^	0.12	0.00	0.68	1.23
		Overweight and obesity	−0.37	0.22	0.23	−0.90	0.16
	Overweight and obesity	Underweight	1.32^*^	0.22	0.00	0.79	1.85
		Norm	0.37	0.22	0.23	−0.16	0.90
4-min run (m)	Underweight	Norm	32.58^*^	7.71	0.00	14.46	50.70
		Overweight and obesity	120.00^*^	13.88	0.00	86.97	153.03
	Norm	Underweight	−32.58^*^	7.71	0.00	−50.70	−14.46
		Overweight and obesity	87.42^*^	13.59	0.00	55.05	119.80
	Overweight and obesity	Underweight	−120.00^*^	13.88	0.00	−153.03	−86.97
		Norm	−87.42^*^	13.59	0.00	−119.80	−55.05

* The difference in means is significant at α ≤ 0.05.

As far as PI is concerned, in the AFA group significant differences were noted in all physical fitness tests between subgroups created in accordance with the aforementioned norms (34). In the control group, however, significant differences were only observed in 3 × 10 m shuttle run and 4-min run (Table 7).

**Table 7:** Physical fitness test results taking into account PI.

**Fitness test**	**PI**	***N***	**Mean**	**SD**	**Standard error**	**95% CI**		**Normality of distribution tests**		***p*-value**
						**Lower**	**Upper**	**Kolmogorov-Smirnov**	**Shapiro-Wilk**	
**AFA group**										
3 × 10 m shuttle run (s)	Underweight (up to 10)	180	8.23	0.61	0.05	8.14	8.32	0.001	0.001	0.001^***^
	Norm (11 do 15)	335	8.34	0.58	0.03	8.27	8.39	0.001	0.001	
	Overweight (16 and above)	12	9.15	0.59	0.17	8.77	9.52	0.200^a^	0.677	
Standing broad jump (m)	Underweight (up to 10)	180	1.84	0.25	0.02	1.80	1.87	0.019	0.038	0.012^*^
	Norm (11 do 15)	335	1.82	0.41	0.02	1.78	1.87	0.001	0.001	
	Overweight (16 and above)	12	1.52	0.19	0.06	1.39	1.64	0.200^a^	0.944	
Medicine ball throw (m)	Underweight (up to 10)	180	7.55	1.82	0.14	7.28	7.81	0.008	0.001	0.021^*^
	Norm (11 do 15)	335	7.95	1.75	0.09	7.76	8.14	0.012	0.001	
	Overweight (16 and above)	12	8.50	1.97	0.57	7.24	9.75	0.200^a^	0.654	
4-min run (m)	Underweight (up to 10)	180	843.56	83.28	6.21	831.30	855.80	0.200^a^	0.058	0.001^***^
	Norm (11 do 15)	335	806.10	94.55	5.17	795.94	816.26	0.001	0.001	
	Overweight (16 and above)	12	667.50	98.27	28.37	605.06	729.94	0.200^a^	0.437	
**Control group**
3 × 10 m shuttle run (s)	Underweight (up to 10)	150	8.97	0.81	0.07	8.84	9.10	0.004	0.001	0.001^***^
	Norm (11 do 15)	491	9.20	0.85	0.04	9.13	9.28	0.001	0.001	
	Overweight (16 and above)	62	9.60	0.72	0.09	9.42	9.78	0.200^a^	0.564	
Standing broad jump (m)	Underweight (up to 10)	150	1.62	0.26	0.02	1.58	1.66	0.200^a^	0.663	0.118
	Norm (11 do 15)	491	1.59	1.02	0.05	1.51	1.69	0.001	0.001	
	Overweight (16 and above)	62	1.37	0.20	0.03	1.32	1.42	0.200^a^	0.288	
Medicine ball throw (m)	Underweight (up to 10)	150	6.24	1.59	0.13	5.99	6.50	0.089	0.059	0.105
	Norm (11 do 15)	491	6.46	1.56	0.07	6.32	6.60	0.001	0.001	
	Overweight (16 and above)	62	6.73	1.71	0.217	6.29	7.16	0.200^a^	0.638	
4-min run (m)	Underweight (up to 10)	150	754.43	101.11	8.26	738.12	770.75	0.038	0.003	0.001^***^
	Norm (11 do 15)	491	718.06	99.44	4.49	709.25	726.88	0.001	0.001	
	Overweight (16 and above)	62	625.01	76.09	9.66	605.69	644.34	0.047	0.001	

a Lower limit of actual significance.

* Statistical significance p-value ≤ 0.05.

*** Statistical significance p-value ≤ 0.001.

In the control group, significant differences were only observed in 3 × 10 m shuttle run and 4-min run; however, *post-hoc* tests showed the significance of differences in standing broad jump as well (Table 7.1).

**Table 7.1:** Significance of differences in physical fitness in relation to PI confirmed by *post-hoc* test (Games-Howell test).

**Dependent variable**			**Difference in means**	**Standard error**	**Relevance**	**95% CI**	
						**Lower**	**Upper**
**AFA group**							
3 × 10 m shuttle run (s)	Underweight	Norm	1.04^*^	0.27	0.01	0.26	1.83
		Overweight	1.08^*^	0.27	0.01	0.30	1.86
		Obesity	0.88^*^	0.27	0.03	0.10	1.65
	Norm	Underweight	−1.04^*^	0.27	0.01	−1.83	−0.26
		Overweight	0.03	0.07	0.96	−0.14	0.21
		Obesity	−0.17^*^	0.06	0.04	−0.33	−0.01
	Overweight	Underweight	−1.08^*^	0.27	0.01	−1.86	−0.30
		Norm	−0.03	0.07	0.96	−0.21	0.14
		Obesity	−0.20^*^	0.06	0.00	−0.35	−0.05
	Obesity	Underweight	−0.88^*^	0.27	0.03	−1.65	−0.10
		Norm	0.17^*^	0.06	0.04	0.01	0.33
		Overweight	0.20^*^	0.06	0.00	0.05	0.35
Standing broad jump	Underweight	Norm	−0.18	0.07	0.06	−0.36	0.01
(m)		Overweight	−0.22^*^	0.07	0.02	−0.41	−0.03
		Obesity	−0.20	0.07	0.06	−0.40	0.01
	Norm	Underweight	0.18	0.07	0.06	−0.01	0.36
		Overweight	−0.04	0.03	0.42	−0.11	0.03
		Obesity	−0.02	0.04	0.97	−0.13	0.09
	Overweight	Underweight	0.22^*^	0.07	0.02	0.03	0.41
		Norm	0.04	0.03	0.42	−0.03	0.11
		Obesity	0.02	0.05	0.95	−0.09	0.14
	Obesity	Underweight	0.20	0.07	0.06	−0.01	0.40
		Norm	0.02	0.04	0.97	−0.09	0.13
		Overweight	−0.02	0.05	0.95	−0.14	0.09
Medicine ball throw (m)	Underweight	Norm	−1.35^*^	0.41	0.02	−2.51	−0.19
		Overweight	−2.26^*^	0.41	0.00	−3.42	−1.10
		Obesity	−2.63^*^	0.40	0.00	−3.79	−1.48
	Norm	Underweight	1.35^*^	0.41	0.02	0.19	2.51
		Overweight	−0.91^*^	0.13	0.00	−1.24	−0.58
		Obesity	−1.28^*^	0.11	0.00	−1.58	−0.99
	Overweight	Underweight	2.26^*^	0.41	0.00	1.10	3.42
		Norm	0.91^*^	0.13	0.00	0.58	1.24
		Obesity	−0.37^*^	0.12	0.01	−0.68	−0.06
	Obesity	Underweight	2.63^*^	0.40	0.00	1.48	3.79
		Norm	1.28^*^	0.11	0.00	0.99	1.58
		Overweight	0.37^*^	0.12	0.01	0.06	0.68
4-min run (m)	Underweight	Norm	−86.77^*^	20.23	0.00	−143.80	−29.74
		Overweight	−75.73^*^	19.98	0.01	−132.32	−19.13
		Obesity	−29.72	19.75	0.46	−85.92	26.49
	Norm	Underweight	86.77^*^	20.23	0.00	29.74	143.80
		Overweight	11.04	8.44	0.56	−10.71	32.79
		Obesity	57.05^*^	7.88	0.00	36.76	77.35
	Overweight	Underweight	75.73^*^	19.98	0.01	19.13	132.32
		Norm	−11.04	8.44	0.56	−32.79	10.71
		Obesity	46.01^*^	7.20	0.00	27.47	64.56
	Obesity	Underweight	29.72	19.75	0.46	−26.49	85.92
		Norm	−57.05^*^	7.88	0.00	−77.35	−36.76
		Overweight	−46.01^*^	7.20	0.00	−64.56	−27.47
**Control group**							
3 × 10 m shuttle run (s)	Underweight	Norm	−0.28^*^	0.05	0.00	−0.41	−0.16
		Overweight and obesity	−0.96^*^	0.09	0.00	−1.18	−0.74
	Norm	Underweight	0.28^*^	0.05	0.00	0.16	0.41
		Overweight and obesity	−0.68^*^	0.09	0.00	−0.89	−0.47
	Overweight	Underweight	0.96^*^	0.09	0.00	0.74	1.18
	and obesity	Norm	0.68^*^	0.09	0.00	0.47	0.89
Standing broad jump	Underweight	Norm	0.05	0.03	0.27	−0.03	0.13
(m)		Overweight and obesity	0.35^*^	0.03	0.00	0.28	0.42
	Norm	Underweight	−0.05	0.03	0.27	−0.13	0.03
		Overweight and obesity	0.30^*^	0.04	0.00	0.21	0.39
	Overweight	Underweight	−0.35^*^	0.03	0.00	−0.42	−0.28
	and obesity	Norm	−0.30^*^	0.04	0.00	−0.39	−0.21
Medicine ball throw (m)	Underweight	Norm	−0.11	0.12	0.62	−0.39	0.17
		Overweight and obesity	−0.06	0.24	0.96	−0.63	0.50
	Norm	Underweight	0.11	0.12	0.62	−0.17	0.39
		Overweight and obesity	0.05	0.23	0.98	−0.49	0.59
	Overweight	Underweight	0.06	0.24	0.96	−0.50	0.63
	and obesity	Norm	−0.05	0.23	0.98	−0.59	0.49
4-min run (m)	Underweight	Norm	49.28^*^	6.72	0.00	33.48	65.07
		Overweight and obesity	171.14^*^	10.95	0.00	145.18	197.10
	Norm	Underweight	−49.28^*^	6.72	0.00	−65.07	−33.48
		Overweight and obesity	121.86^*^	10.11	0.00	97.81	145.92
	Overweight	Underweight	−171.14^*^	10.95	0.00	−197.10	−145.18
	and obesity	Norm	−121.86^*^	10.11	0.00	−145.92	−97.81

* The difference in means is significant at α ≤ 0.05.

Statistical significance of the variation in physical fitness concerning body mass indices, demonstrated by ANOVA analysis, was further confirmed by the Games-Howell *post-hoc* test (Table 8).

**Table 8:** Significance of differences in physical fitness in relation to body mass indices confirmed by *post-hoc* test (*multiple comparisons*).

**Dependent variable**			**Difference in means**	**Standard error**	**Relevance**	**95% CI**	
						**Lower**	**Upper**
Overall fitness test score	Underweight	Norm	7.516	3.719	0.108	−1.21	16.24
Total		Overweight and obesity	62.074^*^	6.091	0.000	47.63	76.52
	Norm	Underweight	−7.516	3.719	0.108	−16.24	1.21
		Overweight and obesity	54.558^*^	6.123	0.000	40.04	69.08
	Overweight and obesity	Underweight	−62.074^*^	6.091	0.000	−76.52	−47.63
		Norm	−54.558^*^	6.123	0.000	−69.08	−40.04
Overall fitness test score	Underweight	Norm	−5.252	4.448	0.465	−15.71	5.20
AFA		Overweight and obesity	73.305^*^	12.113	0.000	42.25	104.36
	Norm	Underweight	5.252	4.448	0.465	−5.20	15.71
		Overweight and obesity	78.558^*^	12.152	0.000	47.44	109.67
	Overweight and obesity	Underweight	−73.305^*^	12.113	0.000	−104.36	−42.25
		Norm	−78.558^*^	12.152	0.000	−109.67	−47.44
Overall fitness test score	Underweight	Norm	9.235	4.197	0.072	−0.63	19.10
Control		Overweight and obesity	35.925^*^	6.851	0.000	19.65	52.20
	Norm	Underweight	−9.235	4.197	0.072	−19.10	0.63
		Overweight and obesity	26.690^*^	6.692	0.000	10.77	42.61
	Overweight and obesity	Underweight	−35.925^*^	6.851	0.000	−52.20	−19.65
		Norm	−26.690^*^	6.692	0.000	−42.61	−10.77

* The difference in means is significant at α ≤ 0.05.

## Discussion

The aim of the study was to assess differences in physical fitness of children and adolescents participating in the AFA program and those not participating in any extra-curricular sports activities.

In all the tests, significantly better results were noted in the AFA group. Similar observations were made by other researchers. In the study of Hazar children taking part in aerobic training (5 times a week for 8 weeks) obtained significantly better results in the majority of tests compared to their untrained peers (35). Also, Ara et al. revealed significant differences in most motor tests between physically active and non-active groups both among girls and boys (36).

In our study, significantly better physical fitness test results were obtained by boys from the AFA group. These findings are in line with the observations of Seccia et al., who revealed significant differences in favor of physically active boys in 4 × 10 m run and standing broad jump (37). Huang et al. (38) reported negative effects of sedentary behaviors on physical fitness levels. In that study, the authors noted significant differences in standing broad jump and 50 m run between girls and boys in favor of the latter group. Slightly different results were presented by Ortega et al., who compared physical fitness levels in girls and boys aged 13. They did not find any significant differences between both genders in standing broad jump and 4 × 10 m shuttle run (39). Also, Ramírez-Vélez et al. did not reveal significant differences in physical fitness tests between both genders (40).

Due to its simplicity, BMI is the most widely used anthropometric tool (41). This index is considered to be a tool for assessing overweight and obesity (42–44). BMI is recognized as a component of physical fitness associated with the health of children and adolescents in different regions of the world (45–47). In the case of anthropometric measurements in children, it is recommended that BMI should be applied together with other anthropometric indices (48). Therefore, Ponderal Index (PI) was also used in the present study (44). This index is more accurate in determining percentage values of body fat than BMI (31). According to Cossio-Bolaños et al., PI may serve as a very useful tool for analyzing physical fitness in adolescents since it is a more accurate index of differences in body mass than BMI (31, 49). Niederer et al. (50) indicate that differences in physical fitness connected with BMI values occur already in pre-school children, and these differences grow larger in older children.

The findings of the current study show that in the AFA group, BMI and PI values are significantly lower. Furthermore, statistical analysis revealed positive correlations of BMI and PI with 3 × 10 shuttle run and 1 kg medicine ball throw in both groups and negative correlations with 4-min run. Only in the AFA group was a negative correlation noted between PI and standing broad jump, which indicates that individuals with higher BMI obtained worse results in the jump test. Ceschia et al. (51) did not show any differences in physical fitness levels between both genders. At the same time, overweight and obese persons demonstrated significantly worse results in the tests of speed, endurance and lower limb strength. Body mass did not affect upper limb power. Conversely, Dumith et al. showed that individuals with normal body mass obtained better results than their counterparts with higher BMI. It was only in the medicine ball throw test that persons with higher BMI scored better (52), which is in line with our findings.

In the OSF test, 3 × 10 m shuttle run constituted the test of speed. In both groups, participants with higher values of BMI and PI had worse results. As for the test of strength (1 kg medicine ball throw), those with higher values of BMI and PI obtained better test results.

In their study, Sacchetti et al. (53) also used standing broad jump and the medicine ball throw. Similar to our findings, they found significant correlations between body mass and test results. Participants who trained regularly also achieved significantly better overall physical fitness test results.

Lower levels of physical fitness in children are associated with a higher risk of obesity as well as cardiovascular and metabolic diseases (54, 55). Well-developed motor abilities may be the factor conditioning a high level of physical fitness that is conducive to increased physical activity in children (56). What is more, physical fitness in childhood is considered to be a significant predictor of current and future health status (2).

Children who achieve early success in a given sport (not necessarily as professionals) are more likely to participate in sports activities and lead an active lifestyle later on (57). Tests that assess physical fitness may be useful in talent identification procedures among children. Also, participation in sports activities chosen based on children's motor abilities increases the likelihood of following an optimal sports career development path in the future. Last but not least, matching an individual anthropometric profile as well as physical and motor fitness of a child with sport-specific characteristics may prevent injuries and early dropout effectively (58).

In the case of gender-related physical fitness differences, Seccia et al. reported significantly better results of boys in 4 × 10 m run (speed test) and in standing broad jump (37). Ortega et al. compared physical fitness levels of girls and boys aged 13 and did not note any significant differences between both genders in standing broad jump and 4 × 10 m shuttle run (39). This regularity was confirmed by Ramírez-Vélez et al. (40). Physical fitness levels are also linked to sedentary activity. Huang et al. (38) revealed a negative influence of sedentary behaviors on physical fitness levels. They noted significant differences between genders (in favor of boys) in power and speed tests.

The findings of our study indicate that physically active children (AFA group) demonstrated higher levels of physical fitness. Numerous researchers confirm correlations between physical activity levels and motor abilities of children (59–64). However, Haga et al. show that these correlations become weaker with age (61). What is interesting, physically active children are more confident with regard to their own motor competences than less active children, which may also influence their sports performance (65).

As many studies indicate (66, 67), the implementation of programs promoting physical activity results in increased overall physical activity particularly among children and adolescents. Verstraete et al. (68) showed that the introduction of a comprehensive physical activity promotion program led to a significant increase in PA engagement.

Correlations between PA levels and PF and motor abilities were also confirmed by Larouche et al. (69). Similar observations were made by Morrison et al. (70), who found correlations between PA and PF as well as between PF and body fat in children aged 6–8.

Undoubtedly, there is a need for implementing research results that show beneficial effects of regular PA in everyday practice. The findings of our study show that it is necessary to introduce physical activity promotion programs for children and adolescents. Such programs should constitute the basis of national health policy aiming at improving physical fitness among young people.

There are some limitations of our study. The research tool used was developed for the purposes of the AFA program. It stemmed from the fact that it was necessary to select tests assessing motor abilities that are fundamental to athletics, i.e., strength, speed, endurance, and power. This work focuses on assessing physical fitness of children and adolescents with the use of the OSF test, a validated tool that has never been applied in any research before. Our findings constitute an introduction to broader analyses of research results obtained within the AFA program regarding physical fitness of children and adolescents in Poland.

## Data availability statement

The raw data supporting the conclusions of this article will be made available by the authors, without undue reservation.

## Ethics statement

The studies involving human participants were reviewed and approved by the Bioethics Committee of Warsaw University of Life Sciences, Faculty of Human Nutrition and Consumer Sciences (Resolution No. 16/2017). Written informed consent to participate in this study was provided by the participants' legal guardian/next of kin.

## Author contributions

JB-K implementation of research and analysis of research material preparation of publications. KZ and MS analysis of research material and preparation of publications. PL preparation of a test sheet. MW implementation of tests and verification of test material. All authors contributed to the article and approved the submitted version.

## Funding

This work has been funded under the Project Physical fitness and physique parameters of children and adolescents participating in the Athletics for All! in comparison with peers not participating in additional physical activity ABNS: PB/2/2022.

## Conflict of interest

The authors declare that the research was conducted in the absence of any commercial or financial relationships that could be construed as a potential conflict of interest.

## Publisher's note

All claims expressed in this article are solely those of the authors and do not necessarily represent those of their affiliated organizations, or those of the publisher, the editors and the reviewers. Any product that may be evaluated in this article, or claim that may be made by its manufacturer, is not guaranteed or endorsed by the publisher.

## Footnotes

^1^Available at: https://www.lekkoatletykadlakazdego.pl/o-programie/zalozenia/o-nas (accessed July 7, 2022).

^2^Available at: https://www.lekkoatletykadlakazdego.pl/dla-trenera/system-szkolenia-sportowego (accessed July 7, 2022).

^3^Available at: https://www.lekkoatletykadlakazdego.pl/dla-trenera/filmy-i-kinogramy/filmy-instruktazowe (accessed July 7, 2022).
